# Flexible Phototransistors on Paper: Scalable Fabrication of PEDOT:PSS Devices Using a Pen Plotter

**DOI:** 10.1002/smsc.202400063

**Published:** 2024-09-16

**Authors:** Yigit Sozen, Gülsüm Ersu, Thomas Pucher, Jorge Quereda, Andres Castellanos‐Gomez

**Affiliations:** ^1^ Materials Science Factory Instituto de Ciencia de Materiales de Madrid (ICMM‐CSIC) E‐28049 Madrid Spain

**Keywords:** organic semiconductor, paper‐based electronics, PEDOT:PSS, Photodetector, phototransistor

## Abstract

Phototransistors are used in plenty of diverse applications such as optical communication systems, light sensors, imaging devices, and biomedical instruments for detecting and amplifying light signals. Herein, an approach for the large‐scale production of low‐cost and flexible phototransistors by integrating the inks of PEDOT:PSS, and graphite with paper, which serves as an ionic conductor material to gate the PEDOT:PSS channel, is proposed. The fabrication of the devices is carried out by sequentially depositing the PEDOT:PSS channel and graphite electrodes onto paper using a benchtop XY plotter. To characterize device‐to‐device variability, 200 devices are fabricated and their electrical and optical properties are statistically analyzed. By performing a detailed characterization on the optical properties under varying wavelength, power, and bias conditions, it is found that devices exhibit good photoresponse across a wide spectrum range. Moreover, devices maintain their photoactive characteristics even when subjected to high mechanical tensile strain, indicating the suitability of these paper‐supported devices for flexible electronic applications. Time and photocurrent magnitude can be tuned via gate voltages applied through the graphite‐based back‐gate configuration.

## Graphical abstract

This study details the development of low‐cost, flexible phototransistors by depositing PEDOT:PSS and graphite inks on paper using benchtop XY pen plotter. A continuous operating process for the large‐batch production of the PEDOT:PSS/graphite devices is demonstrated, which shows good optoelectronic features. The devices’ electronic and optical performances are tuned via gate voltage, making them suitable for flexible electronics.

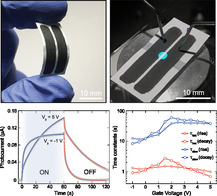

## 1 Introduction

Printed electronics is a promising avenue to produce low‐cost devices with a lower electronic waste footprint as compared with silicon‐based electronics.^[^ ^1^, ^2^ ^]^ Unlike the complex manufacturing steps (such as photolithography, vacuum deposition, and etching) utilized in silicon‐based device fabrication, printing techniques rely on the direct deposition of solution‐processable inks of organic or inorganic materials^[^ ^3^, ^4^, ^5^, ^6^ ^]^ onto a desired substrate. To date, various printing techniques have been reported, such as inkjet printing,^[^ ^7^, ^8^, ^9^ ^]^ gravure printing,^[^ ^10^, ^11^, ^12^ ^]^ and screen printing.^[^ ^13^, ^14^ ^]^ These deposition techniques have not only provided cost‐effectiveness by reducing material consumption, but also the dependence on rigid substrates such as silicon and glass, allowing researchers to extend device fabrication to mechanically stretchable and flexible substrates such as paper,^[^ ^15^, ^16^, ^17^, ^18^ ^]^ plastic,^[^ ^19^, ^20^, ^21^ ^]^ and textiles.^[^ ^22^, ^23^ ^]^ The combination of these printing techniques with the flexible substrates allows for the fabrication of flexible, lightweight, and thin forms of electronic and optoelectronic devices and meeting the requirements of new applications such as wearable electronics, biosensors, and real‐time health monitoring.^[^ ^22^, ^24^, ^25^, ^26^ ^]^ Moreover, these printing processes are feasible for high‐throughput and large‐volume manufacturing, making them viable for the commercial applications of flexible electronic devices.^[^ ^27^, ^28^, ^29^ ^]^

Paper is emerging as a relatively inexpensive, lightweight, biodegradable, and easily accessible substrate compared to other flexible substrates.^[^ ^30^, ^31^ ^]^ In fact, paper is becoming an attractive candidate as a substrate for cost‐effective production of electronic devices.^[^ ^32^, ^33^, ^34^, ^35^, ^36^ ^]^ Several studies have reported that ink‐depositing approaches are feasible to form precise patterns of nanostructured inks of materials on paper.^[^ ^15^, ^37^, ^38^, ^39^, ^40^ ^]^ However, most works are based on the use of paper substrates with a specialized surface treatment to ensure good printability and these substrates are expensive and less biodegradable than standard untreated papers.^[^ ^16^, ^17^, ^41^, ^42^ ^]^ Moreover, device‐to‐device variability is scarcely investigated in previous literature, while being a crucial factor for the potential applicability of paper‐based devices. Therefore, some key aspects still need to be investigated in detail, to verify the strength of paper for large‐scale, low‐cost, and biodegradable electronics.

In our previous study, we presented the benchtop XY pen plotter setup, which is a simple and inexpensive process for depositing solution‐processable inks on standard copy paper (without need of any further surface treatment) to fabricate paper‐based devices.^[^ ^43^ ^]^ In this study, we demonstrated a continuous operating process with the same method for the large‐batch production of poly(3,4‐ethylenedioxythiophene) polystyrene sulfonate (PEDOT:PSS) and graphite‐based phototransistors. The fabrication process is explained in detail, starting with the production of photoresistors and extending to the manufacturing of a phototransistor. The fabrication method is easy to process and relies on device patterning onto an A4 size paper substrate with the consecutive deposition of the corresponding materials’ inks via marker pens filled in with the ink of the desired material. Here, by fabricating a large number of devices, we present a detailed statistical and optoelectronic analysis and discuss the key functionality of PEDOT:PSS/graphite devices by operating them as phototransistors. Overall, our work presents a simple, low‐cost, and fully ecological route for the mass production of paper‐based phototransistors.

## 2 Results

### 2.1 Fabrication Method and Structural Characterization

**Figure 1:**
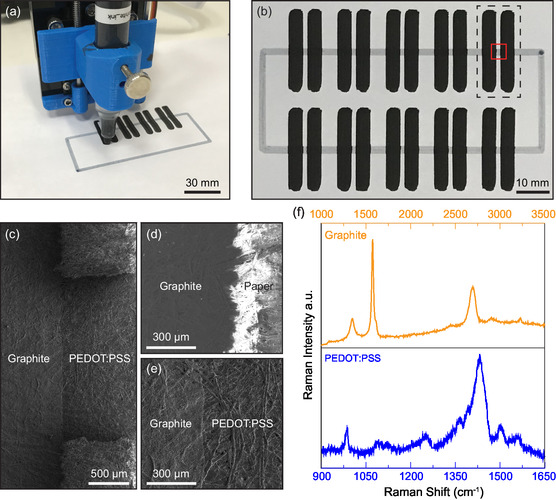
a) Image taken during the fabrication process of PEDOT:PSS/graphite‐based photoresistors on paper using a pen plotter. b) Optical image of an as‐fabricated device batch containing ten devices. The black dashed lines represent the single device, and the red solid lines indicate the location of the PEDOT:PSS‐based photoactive channel between the long graphite source–drain contacts. c,d and e) SEM images acquired from different ink‐covered regions. f) Raman spectra of the graphite and PEDOT:PSS ink‐covered regions.

First, we consider photoresistor fabrication by depositing PEDOT:PSS as a light‐sensitive channel and graphite as electrodes. After filling the broad‐tip refillable marker pen with the corresponding inks of PEDOT:PSS and graphite (see Experimental Section), we fabricated devices by performing successive pattern plots of each material on standard copy paper. First, a rectangular pattern of PEDOT:PSS was plotted on the paper, which was later dried by heating it on a hot plate at 60 °C for 5 min. Long graphite electrodes were then plotted and the drying process was repeated. Note that the reverse pattern plotting could not be implemented as we found that PEDOT:PSS printing tends to damage the graphite electrodes. **Figure** **1**a shows an image acquired during the fabrication process of a batch of photodetectors. Each single batch (Figure 1b) contains a total of ten PEDOT:PSS/graphite devices and only takes few minutes to produce. The measured channel lengths for devices vary between 0.7 and 0.9 mm, the channel widths vary between 2.8 and 3.0 mm. After fabrication, a single device can be isolated from the batch using a sharp razor blade or scissors.

Next, we analyzed the surface morphology of the devices using scanning electron microscopy (SEM). The image in Figure 1c shows a low‐magnification image near one of the electrodes, indicating that regions covered with different inks can be distinguished through the contrast difference due to differences in conductivity. Figure 1d,e shows the images taken from graphite–paper and PEDOT:PSS–graphite interfaces, respectively.

We extended our characterization by obtaining Raman spectra of ink‐covered regions. The corresponding Raman spectra are shown in Figure 1f. For graphite, the most prominent Raman‐active mode, the G band, is observed at 1576 cm^−1^, while the D and G’ bands are located at 1348 and 2696 cm^−1^, respectively.^[^ ^44^, ^45^, ^46^ ^]^ The Raman spectrum acquired at the PEDOT:PSS‐covered regions shows a prominent peak at 1431 cm^−1^, which originates from the symmetric stretching resonant mode (*C*_α_ = *C*_β_). Less prominent peaks are also present at 987, 1252, 1366, 1502, and 1559 cm^−1^, in good agreement with previous reports for this material.^[^ ^47^, ^48^ ^]^

### 2.2 Electrical Characterization

**Figure 2:**
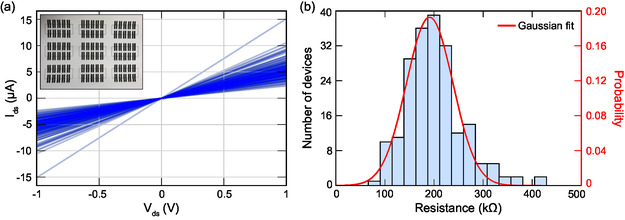
a) Current–voltage curves collected from 200 PEDOT:PSS/graphite devices. The inset figure shows the image of nine device batches (90 devices) plotted on paper. b) The histogram graph represents the distribution of calculated resistance values of the fabricated devices.

We fabricated and characterized the electrical properties of 200 PEDOT:PSS/graphite devices in terms of their current–voltage (*I–V*) characteristics. **Figure** **2**a shows an assembly of all acquired *I–V* curves, with Ohmic‐type behavior. The inset of the corresponding figure shows a single sheet of A4 standard copy paper containing nine device batches (90 devices). Following the *I–V* measurements, we obtained the resistance values from the slopes of the *I–V* curves. Figure 2b shows that the resistance data is clustered around a central point and can be fit to a Gaussian distribution. Accordingly, the mean of the distribution is localized at 191 kΩ, which indicates a low channel resistivity. The standard deviation was calculated to be 47 kΩ, accounting for roughly 25% of the total device resistance. Such device‐to‐device variation may be due to the heterogeneous composition of copy paper, which prevents the formation of a smooth interface with the ink.

We also investigated the variation in channel resistance by increasing the distance between two graphite electrodes following a transfer length method approach. The current versus voltage characteristics were obtained for channel lengths ranging from 0.9 to 5.5 mm. Extracting the resistance values from each *I–V* curve and representing them as a function of the channel length show that resistance values follow a linear increase with decreasing channel length. By performing a linear regression to the resistance dataset, we obtained a slope of 89.6 kΩ mm^−1^, with a *y*‐intercept located below 0, indicating a negligible contact resistance (within the experimental uncertainty). Detailed information can be found in the Supporting Information (Figure S1, Supporting Information).

### 2.3 Optoelectronic Characterization

**Figure 3:**
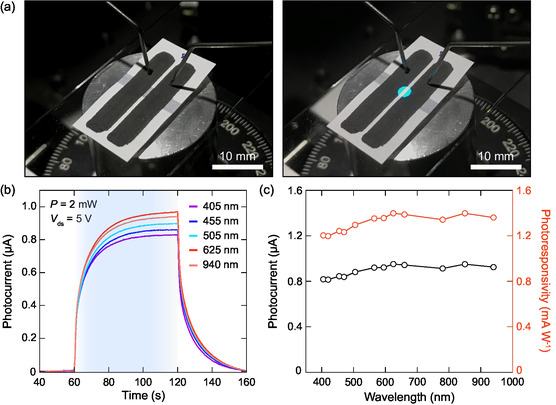
a) Images of a PEDOT:PSS/graphite photodetector taken in the dark state and upon illumination (*λ* = 470 nm). b) Evaluation of current with time under light excitation for different wavelengths ranging from the visible to near‐infrared (405–940 nm). c) Calculated photocurrent and photoresponsivity values with varying wavelength excitations.

After the basic structural and electrical characterization, we investigated the photodetection performance of PEDOT:PSS/graphite‐based paper photodetectors by studying the photocurrent generation under varying wavelength, power, and bias voltages. The images of the device in the dark and under illumination are shown in **Figure** **3**a. Wavelength‐dependent photocurrent measurements were carried out using high‐power fiber‐coupled light emitting diode (LED) sources with 12 different wavelengths covering the range from 405 to 940 nm.^[^ ^49^ ^]^ Accordingly, the device was excited with a light beam of 2 mW power for each wavelength with a spot size of 1.25 mm radius covering 82% of the active channel area. We performed wavelength‐dependent time‐resolved photocurrent measurements, where the drain–source current was collected as a function of time after the LED source was turned ON (see Figure 3b). After 60 s of irradiation, the LED was turned OFF for 60 s, at which point the wavelength value was changed to repeat the same process continuously. As shown, when the device was exposed to illumination with light sources ranging from violet to near‐infrared, we observed an enhancement in electrical current, showing the suitability of the device to detect light over a wide spectral range. We calculated the photocurrent values as the difference between the current values obtained at dark and bright states (*I*_ph_ = *I*_bright_—*I*_dark_), and the results are represented in Figure 3c. Within the considered spectral range, the photocurrent shows no significant change for wavelengths up to 940 nm. In order to quantitatively determine the light sensitivity of the device, we further calculated the responsivity (*R*) using the equation

(1) $$R=\frac{I_{\text{ph}}}{P}\times \frac{A_{\text{spot}}}{A_{\text{active}}}$$

where *I*_ph_ is the generated photocurrent, *P* is the power of the incident light, *A*_spot_ is the total area of the focused light spot, and *A*_active_ is the area of the illuminated active channel between the electrodes. We represented the photoresponsivity values as a function of the wavelength of the incident radiation in Figure 3c, which indicates a variation between 1.2 and 1.4 mA W^−1^.

**Figure 4:**
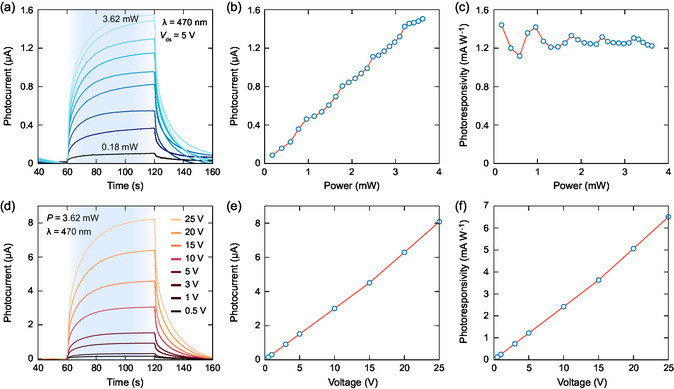
a) The evaluation of current–time characteristics under increasing light (470 nm) power, and corresponding b) photocurrent and c) photoresponsivity values. d) The evaluation of current–time characteristics with varying bias voltages and corresponding e) photocurrent and f) photoresponsivity values.

We further investigated the power dependence of the photocurrent by increasing the illumination power from 0.18 to 3.62 mW. **Figure** **4**a shows the photocurrent signal over time under different incident light powers. As shown, the increase in the irradiation power results in a larger photocurrent signal. Figure 4b shows the calculated photocurrent values as a function of the power, revealing that photocurrent exhibits a linear dependence on the excitation power across the considered power range. This implies nondependency of photoresponsivity to the illumination power (see Figure 4c). With the highest power excitation, the photocurrent reached a value of 1.51 μA, and the mean value for the photoresponsivity through the considered power range was calculated to be 1.27 mA W^−1^. To extend our characterizations, we performed photocurrent measurements by operating the device under different bias voltages (see Figure 4d). As shown in Figure 4e, increased bias voltage led to an increase in the photocurrent due to the increased separation efficiency of the photogenerated charge carriers. When the device was operated at the highest considered voltage (25 V), we determined a photocurrent of 8.1 μA, and the photoresponsivity of the device reached to 6.5 mA W^−1^ (see Figure 4f), which is higher as compared to the previously reported photodetectors fabricated through the other conventional printing methods on paper.^[^ ^19^, ^50^, ^51^, ^52^, ^53^ ^]^

From the photocurrent versus time traces while switching ON/OFF the illumination, we can conclude that the photoresponse mechanism can result from two different phenomena. The fast response immediately after excitation is followed by a more dominant slow response which can result from the photogating or bolometric effect. However, the linear dependence of the photocurrent to incident light power eliminates the possibility of a photogating effect as it typically follows sublinear dependence on power because of the filling of the midgap trap states at high densities of photogenerated charge carriers.^[^ ^54^, ^55^, ^56^ ^]^ On the other hand, the photoresponse of the device is not wavelength selective, which is also a typical feature of the bolometers. In that case, the slow photoresponse can be attributed to the bolometric effect and probably due to the low thermal conductivity of paper substrate that prevents from a fast heat sinking after the illumination is switched OFF.

**Figure 5:**
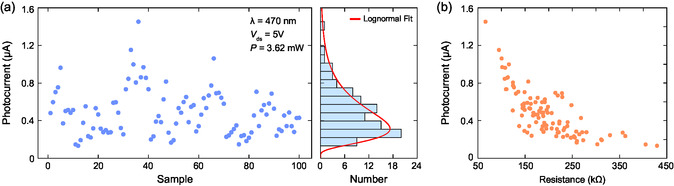
a) Statistical analysis of photocurrent measurements performed on a sample set consisting of 100 devices, using a 470 nm light source at 3.62 mW power with a drain–source voltage of 5 V. The plots present the variation and distribution in the photocurrent values over the measured devices. b) The variation in photocurrent as a function of the channel resistance.

To obtain the device‐to‐device variation in photocurrent generation, we performed photocurrent measurements by illuminating individual devices under 470 nm light with an excitation power of 3.62 mW, with a bias voltage of 5 V. The sample set of 100 devices was randomly selected from the total set of 200 devices. The statistical distribution for the collected photocurrent data is shown in **Figure** **5**a. As shown, the photocurrent values are distributed over a wide spectrum. When the collected dataset was fit to the log‐normal distribution, the median of the distribution was found to be 0.3 μA. The plot in Figure 5b shows the relation between channel resistance and photocurrent. It is seen that the increased channel resistance in a device led to less photocurrent generation. This may be due to reduced carrier transport at high resistance, which prevents the photogenerated carriers from flowing through the channel.

Since flexibility is one of the key characteristics of the article, we further investigated how external strain affects the photocurrent characteristics of a device. To induce a uniaxial tensile strain ranging from 0 to 1%, we used 3D‐printed stages with different‐curvature radii. The strain (*ε*) is determined using the formula *ε* = *h*/2*R*, where *h* is the thickness of the substrate (100 μm for the paper) and *R* is the radius of the curvature.^[^ ^57^ ^]^ Afterward, consecutive time‐resolved photocurrent measurements were carried out by clamping a device on these stages with scotch tape (see Figure S2a, Supporting Information). After straining to 1%, photoresponsivity persisted and a slight drop in photocurrent was observed. This drop was permanent as we did not observe a full recovery of the initial photocurrent value when strain was released. The plot represented in Figure S2b, Supporting Information, shows the photocurrent values as a function of the strain.

### 2.4 Fabrication and Characterization of Back‐Gated Phototransistors

**Figure 6:**
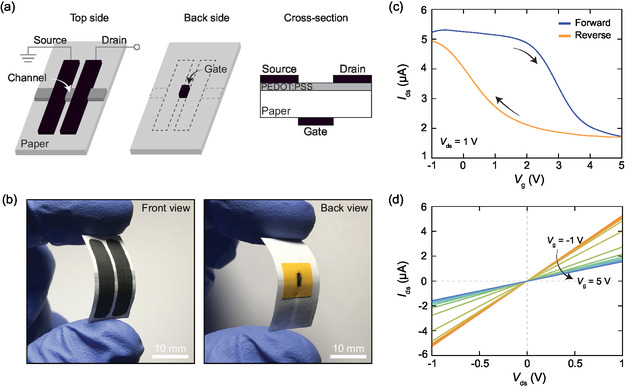
a) Top, side, and cross‐sectional schematic views of back‐gated PEDOT:PSS phototransistor structure. b) Front and back views from the final structure of the flexible back‐gated PEDOT:PSS/graphite phototransistor. c) Source–drain current (*I*_ds_) as a function of the gate voltage (*V*_g_), under a source–drain voltage (*V*_ds_) of 1 V. d) Source–drain current (*I*_ds_) versus source–drain voltage (*V*_ds_) characteristics while gradually changing the gate voltage (*V*_g_) from −1 to 5 V.

As a next step, we investigated the charge transport characteristics of PEDOT:PSS/graphite devices using them as field‐effect transistors. To modulate the current flow through the channel of initially fabricated resistors, we used the paper substrate as an ionic conductor to generate an electric field through an electric double‐layer effect. To generate an electric field across the paper, we considered graphite‐based back‐gate electrode structure by plotting the back side of the channel with the graphite ink. **Figure** **6**a shows the schematic representation of the transistor structure from different perspectives, including the top, side and cross‐sectional views. Figure 6b shows pictures of the front and back views of a PEDOT:PSS/graphite transistor. The detailed images of the final device structure can be seen in the supporting information (Figure S6, Supporting Information). By applying a gate voltage through this back‐gate electrode, we induce cation–anion pair formation due to the existence of the calcium carbonate fillers and hydroxyl groups in the paper.^[^ ^58^, ^59^, ^60^, ^61^ ^]^ This ionic polarization in the paper leads to modulation of channel conductivity resulting from an accumulation of ions near the channel interface.

The electrical characteristics of the fabricated transistors were investigated by ranging gate voltages from −1 to 5 V. We avoided increasing the gate voltage to more negative values than −1 V to prevent a permanent drop in the drain–source current. Such changes in the electrical characteristics of the devices are most likely due to a chemical reaction between the ions accumulated at the interface and the channel material. The transport measurements were carried out under ambient conditions by modulating the gate across the considered voltage range. Owing to the slow change in channel conductivity under the gate, a noticeable amount of waiting time should be considered before sweeping the gate. The slow response can be attributed to the slow ion migration resulting from the ionic nature of the gating. In our measurements, we found that a sweep rate of 1.6 mV s^−1^ (0.01 V every 6 s) is adequate to carry out a proper transfer curve measurement. Figure 6c shows the transfer curve with a fixed source–drain voltage (*V*_ds_ = 1 V) and indicates p‐type behavior of the PEDOT:PSS channel. From the data represented in Figure 6c, we can extract the mobility of the field‐effect transistor using the formula

(2) $$\mu =\;\frac{dI_{\text{ds}}}{dV_{g}}\;\frac{L}{W}\;\frac{1}{C_{G}V_{\text{ds}}}$$

where *I*_ds_, *V*_ds_, and *V*_g_ are the source–drain current, source–drain voltage, and gate voltage, *L* and *W* are channel length and width of the transistor, and *C*_g_ is the capacitance of the regular paper. Using the previously reported capacitance density for standard office paper, *C*_g_ = 4 ×10^−8^ F cm^−2^,^[^ ^62^ ^]^ one can get a rough estimate of the mobility as ≈13 cm^2^ V^−1^ s^−1^. This value is in the same order as previously reported ionic‐gated PEDOT:PSS organic transistors^[^ ^63^ ^]^ but note that our value is only an estimate as we did rely on the reported capacitance density values because of the lack of the devoted setup to carry out this measurement accurately. Figure 6d gives the variation in source–drain characteristics for the PEDOT:PSS/graphite device under varying gate voltages (from −1 to 5 V in 0.5 V steps). Note that we waited for 400 s before starting the next measurement to allow for the system to stabilize. The device maintains a linear current–voltage characteristic over the considered gate range, indicating the persistence of the Ohmic contact between two materials. When we repeated the transistor measurements for two additional devices, we obtained identical behavior under the same operating conditions, demonstrating uniform gate‐dependent properties for PEDOT:PSS/graphite transistors (see Figure S7, Supporting Information).

### 2.5 Gate‐Dependent Optoelectronic Measurements

**Figure 7:**
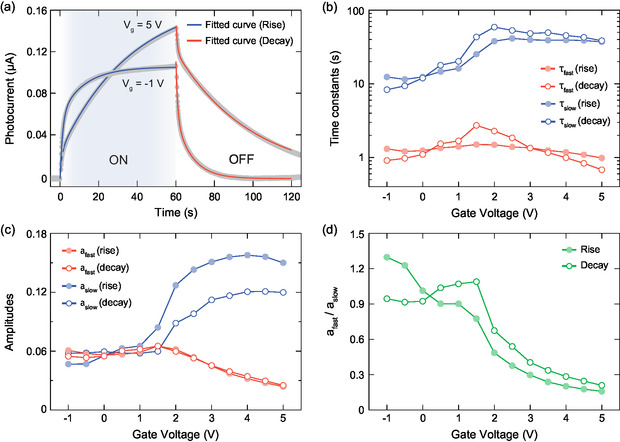
a) Photocurrent time traces obtained at two different gate voltages. The measurement is performed using 470 nm LED source with 3.62 mW power, under a bias voltage of 1 V. Blue and red curves are two‐term exponential fits to the rising and decaying part of the corresponding data. The gate‐dependent change is shown for b) time constants and c) amplitudes and derived from two‐term exponential fit performed for rising and decaying signals. d) The ratio of the amplitudes.

We also investigated the variation in photocurrent characteristics in response to the applied gate voltage. For this purpose, successive time‐resolved photocurrent measurements were carried out with varying gate voltages. In a set of measurements, the gate voltage was swept from −1 to 5 V with a step of 0.5 V. Due to the slow response time originated from ionic gating, the waiting periods before and after photocurrent measurements were extended to 300 s, which introduced a significant change in the channel conductivity to observe the complete effect of gating on photocurrent characteristics. **Figure** **7**a shows time‐resolved photocurrent cycles obtained under two opposite gate voltages. Interestingly, when the gate was swept toward more positive values, the photoresponse of the device became sizeably slower. Moreover, we also observed that positive gate voltages led to higher photocurrent values. Negative gate voltages, on the other hand, yield lower photocurrent values and faster response. The evaluation of the photocurrent as a function of the gate voltage is represented in Figure S8, Supporting Information.

To perform a comparative analysis of variations in the physical components of a photocurrent signal under varying gate voltage, we used a two‐term exponential fit by considering the rising and decaying parts of the photocurrent signals. This theoretical framework allows for detailed examination of effects supporting the photoresponse mechanism. The two‐term exponential models for the rising and decaying signal are given

(3) $$I_{\text{rise}}\left(t\right)=\sum_{n=1}^{2}a_{n}\left(1-e^{-{\text{t/}\tau }_{n}}\right)$$

(4) $$I_{\text{decay}}\left(t\right)=\sum_{n=1}^{2}a_{n}\left(e^{-{\text{t/}\tau }_{n}}\right)$$

where $a_{n}$ is the peak amplitude of the fitted function, $\tau_{n}$ is the time constant, corresponding to the response time of the device.

Figure 7a shows an excellent fit of the experimental data to the two‐term exponential model, indicating that the photocurrent generation is mainly due to two processes: one faster and one slower. After the two‐term exponential fit, the time constants (amplitudes) for fast and slow components were separately examined by labeling them as $\tau_{\text{fast}}$ ($a_{\text{fast}}$) and $\tau_{\text{slow}}$ ($a_{\text{slow}}$), respectively. In Figure 7b,c, the acquired time constants and amplitudes for rising and decaying signals are represented as a function of the gate voltage. In each signal, $\tau_{\text{slow}}$ is consistently ten times larger than $\tau_{\text{fast}}$, and both components display distinct behavior as the gate voltage varies. While $\tau_{\text{fast}}$ remains relatively stable during the rising signal within the considered voltage range, the change in this parameter is more pronounced in the decaying part, such that it exhibits a threefold increase when the gate voltage is swept up to 2 V, which is followed by a fourfold decrease afterward. On the other hand, the effect of the gate was more prominent on $\tau_{\text{slow}}$, which showed a threefold and sevenfold increase in rising and decaying signal, respectively, as the gate was swept up to around 2 V. From 2 to 5 V, $\tau_{\text{slow}}$ showed a negligible decrease in rising signal in contrast to decaying part where the drop was more noticeable. Overall, an increase in positive gate values resulted in slower response due to much larger increase in $\tau_{\text{slow}}$ compared to $\tau_{\text{fast}}$.

Interestingly, as shown in Figure 7c, the amplitude of the fast and slow components of the photocurrent ($a_{\text{fast}}$ and $a_{\text{slow}}$) are comparably similar from negative values up to 1.5 V but the slow component starts to rise and the fast component decays for more positive gate values. Figure 7d shows the ratio of the amplitudes to provide information about the overall behavior of the device with varying gate voltage. Within the gate voltages of −1 and 1.5 V, the ratio between $a_{\text{fast}}$ and $a_{\text{slow}}$ is close to 1, which indicates the balanced contribution of fast and slow mechanisms to the photocurrent. However, higher positive gate voltages led to a gradual decrease in ratio, indicating that the slow mechanism becomes more dominant. When these measurements were replicated for two different devices, we found a similar trend across the same gate range (see Figure S9 and S10, Supporting Information). Although the microscopic mechanism that explains the observed gate‐tunable photoresponse is still unclear, these results illustrate how one can tune the magnitude of the photoresponse and the device speed using the gate voltage, increasing the functionality of these devices with respect to more basic paper‐based photodetectors.

While our devices show two photocurrent generation mechanisms, one fast ($\tau_{\text{fast}}\approx 1\text{ s}$) and one slow ($\tau_{\text{slow}}\approx 10s$), whose contributions can be controlled with the gate voltage, other PEDOT:PSS‐based photodetectors have shown a photocurrent generation mostly dominated by the slow response mechanism. A previous study has introduced Se/PEDOT:PSS hybrid junction photodetectors on the flexible PET substrate, the response time was ≈9 s.^[^ ^64^ ^]^ Improved response time has been achieved in hybrid devices modifying PEDOT:PSS thin films with fullerene (*C*_60_) molecules showing rise/fall times in the 6–7 ms range.^[^ ^65^ ^]^ Also, the combination of PEDOT:PSS with Si, on the other hand, provides devices with response times within the milli‐ and microsecond range, but those devices are on rigid silicon substrates and lack of the advantages of flexible and biodegradability of paper substrates.^[^ ^66^, ^67^ ^]^

## 3 Conclusions

In this article, we have presented a robust procedure for the large‐scale fabrication of ink‐based PEDOT:PSS/graphite phototransistors on paper, utilizing a benchtop XY plotter. Statistical analyses conducted on a large number of devices have demonstrated moderate device‐to‐device variability, affirming the reproducibility of our method. Detailed optoelectronic characterizations have revealed the high photoresponse of PEDOT:PSS/graphite photodetectors across the entire visible spectrum, attributed to the bolometric effect. Strain‐dependent measurements revealed that devices maintain photoactivity even at high uniaxial strains like 1%. Furthermore, we have shown the suitability of the device as a field‐effect transistor by modulating the channel conductivity within a low gate voltage range using paper as a back‐gate dielectric. Notably, sweeping the gate field enables precise control over the amplitude and response time of two distinct photocurrent mechanisms within the decay and rising photocurrent signals. In conclusion, our results demonstrate that the deposition of solution‐processable materials on paper with an automated benchtop plotter is consistent and feasible for the mass fabrication of low‐cost and biodegradable electronics.

## 4 Experimental Section

4.1

4.1.1

##### Resource Availability: Lead Contact

Further information and requests for resources should be directed to and will be fulfilled by the lead contact, Andres Castellanos‐Gomez (andres.castellanos@csic.es).

##### Materials Availability

All materials generated in this study are available from the lead contact upon reasonable request.

##### Materials

PEDOT:PSS ink was purchased from Ossila (M122), and graphite ink was prepared by dissolving a commercial ink purchased from Aquadag Graphite (Agar, G303E) in DI water (1:1 v/v). Inks were deposited on standard office paper (MultiOffice stress‐free paper, 80 g m^−2^) with a thickness of 100 μm. The vinyl masks used to deposit back gate electrodes were prepared by cutting an adhesive vinyl sheet (Ohuhu permanent adhesive‐backed vinyl sheets) using a benchtop smart cutting machine (Cricut Maker 3). Strain stages were fabricated using ELEGOO Mars 2 resin 3D printer.

##### Plotting Process

The plotting process was carried out by means of a Baugger desktop plotter machine using broad‐tip markers (Magiin empty refillable marker pen) containing PEDOT:PSS and graphite inks. The desired device patterns were created using a vector graphics editor (Inkscape), and their raster images were loaded into the plotter software for plotting. Printing speeds between 1400 and 1200 mm min^−1^ were found to be suitable for carrying out an efficient ink deposition during plotting.

##### Back‐Gate Fabrication

To pattern the back‐gated transistor structure, first, we fabricated a vinyl mask with a small rectangular window with a length of 3 mm and a width of 1 mm. The size of the rectangular window was chosen approximately close to the channel size to prevent leakage between the source–drain and gate electrodes. Later, the vinyl mask was adhered to the back side of the device by carefully aligning the window with PEDOT:PSS active channel. Later the gap was covered with graphite ink to form the bottom gate electrode and annealed on a hot plate at 60 °C for 5 min to let it dry. As a final step, the graphite‐covered gate window was contacted with copper tape and a wire for external connection.

##### Electronic and Optical Measurements

To perform electronic and optical characterizations, source–drain electrodes of PEDOT:PSS/graphite phototransistors were connected to the Keithley 2450 source measure unit with a homebuilt probe station. All measurements were carried out in air at room temperature. Fiber‐coupled LED sources (Thorlabs, MxxxFy series) were used as a light source to perform power, bias, and wavelength‐dependent measurements. The end of the multimode fiber was coupled to a collimator to produce a light spot with a radius of 1.25 mm on the sample. For power‐dependent measurements, the LED source was operated in modulation mode and controlled with a single‐channel programmable benchtop power supply (Tenma 72‐2715) to vary the incident light power. The light power was measured using an optical power meter (Thorlabs, PM100D) coupled to a photodiode power sensor (Thorlabs, S120C). The gate‐dependent electronic and optoelectronic measurements were performed by pairing two programmable bench‐top power supplies (Tenma 72‐2715) in parallel to provide gate modulation in negative and positive voltage range.

##### Raman Characterization

Raman measurements for graphite and PEDOT:PSS materials on paper were performed under ambient conditions using a confocal Raman microscope (MonoVista CRS+, Spectroscopy & Imaging GmbH). The optical excitation was provided by a 532 nm line of a continous wave (CW) solid‐state laser with an incident light power of 0.5 mW through a 100× magnification microscope objective (NA = 0.9). The 300 and 1500 lines/mm grating were used for graphite and PEDOT:PSS, respectively.

##### Scanning Electron Microscopy

The surface morphology of the inks on paper was examined through the FEI Nova NanoSEM 230 FE‐SEM.

##### Durability Tests

We investigated the optoelectronic performance of nongated photodetectors under various environmental conditions like air exposure, temperature, and light irradiation to understand how these factors change the durability of devices. To investigate the long‐term stability of four PEDOT:PSS/graphite devices, we left them under atmospheric conditions for 20 days. During this time, we checked the evolution of the electronic and optical properties by performing current–voltage and time‐resolved photocurrent measurements. As shown in Figure S3, Supporting Information, conductivity and photocurrent in each device decreased over time. After a significant decrease in photocurrent in each device during the first 5 days, this drop became more gradual later. We determined the percentage of decrease in the photocurrent, which ranged between 59% and 74%. This drop in performance indicated that atmospheric conditions can affect the device's performance. This mainly resulted from the hygroscopicity of PEDOT:PSS, which means that it absorbs moisture from the air and can degrade in time because of oxidation. Therefore, further study is needed to enhance the stability of PEDOT:PSS/graphite‐based photodetectors. Next, we investigated the durability of the photodetectors to the light exposure by performing a continuous photoswitching for 50 cycles. Figure S4a, Supporting Information, shows the whole ON/OFF illumination cycles acquired from the time‐resolved photocurrent measurement. Figure S4b, Supporting Information, includes the photocurrent values extracted from each ON/OFF cycle and a histogram to see the photocurrent distribution. As shown, photocurrent values showed low variability, indicating the durability of the device for long‐term operation under illumination. For temperature stability, we analyzed the photocurrent as a function of the temperature. We performed time‐resolved photocurrent measurements by increasing the temperature from 23 to 60 °C. Figure S5a, Supporting Information, shows that device shows a decrease in photocurrent and exhibits electrical noise with increasing temperature. The photocurrent measured as 0.38 μA at room temperature decreased to 0.10 μA at 60° (see Figure S5b, Supporting Information), a total decrease of 67%, which was a significant drop in a narrow temperature range. However this drop is reversible since we obtained same photocurrent value after cooling the device to room temperature. Consequently, temperature should be considered as an important factor that can affect the performance of the PEDOT:PSS/graphite photodetectors.

## Conflict of Interest

The authors declare no conflict of interest.

## Author Contributions

**Yigit Sozen**: Data curation (lead); Formal analysis (lead); Investigation (lead); Methodology (lead); Writing—original draft (lead); Writing—review & editing (equal). **Gülsüm Ersu**: Data curation (supporting); Formal analysis (supporting); Investigation (supporting); Methodology (supporting). **Thomas Pucher**: Data curation (supporting); Formal analysis (supporting); Investigation (supporting); Methodology (supporting). **Jorge Quereda**: Formal analysis (supporting); Supervision (supporting); Writing—review & editing (equal). **Andres Castellanos‐Gomez**: Conceptualization (lead); Funding acquisition (lead); Methodology (supporting); Project administration (lead); Resources (lead); Supervision (lead); Writing—original draft (supporting); Writing—review & editing (equal).

## Supporting information

Supplementary Material

## Data Availability

The data that support the findings of this study are available from the corresponding author upon reasonable request.

## References

[1] K. Suganuma , in Introduction to Printed Electronics, Springer Science & Business Media, New York 2014.

[2] Y. Khan , A. Thielens , S. Muin , J. Ting , C. Baumbauer , A. C. Arias , Adv. Mater. 2020, 32, 1905279. 10.1002/adma.201905279 31742812

[3] Y. Aleeva , B. Pignataro , J. Mater. Chem. C 2014, 2, 6436.

[4] F. Bonaccorso , A. Bartolotta , J. N. Coleman , C. Backes , Adv. Mater. 2016, 28, 6136. 27273554 10.1002/adma.201506410

[5] C. Backes , A. Bianco , C. Casiraghi , F. Galembeck , R. K. Gupta , M. C. Hersam , A. R. Kamali , M. Kolíbal , V. Kolosov , V. Kumar , W. H. Lee , N. Martsinovich , M. Melchionna , K. Müllen , A. Oyarzun , V. Palermo , M. Prato , P. Samori , S. Sampath , A. Silvestri , D. Sirbu , R. Sui , A. Turchanin , C. Wetzl , I. A. Wright , Z. Xia , X. Zhuang , Faraday Discuss. 2021, 227, 141. 33877206 10.1039/d1fd90002a

[6] K. Parvez , R. Li , S. R. Puniredd , Y. Hernandez , F. Hinkel , S. Wang , X. Feng , K. Müllen , ACS Nano 2013, 7, 3598. 23531157 10.1021/nn400576v

[7] M. Singh , H. M. Haverinen , P. Dhagat , G. E. Jabbour , Adv. Mater. 2010, 22, 673. 20217769 10.1002/adma.200901141

[8] Z. Zhan , J. An , Y. Wei , V. T. Tran , H. Du , Nanoscale 2017, 9, 965. 28009893 10.1039/c6nr08220c

[9] A. Moya , G. Gabriel , R. Villa , F. J. del Campo , Curr. Opin. Electrochem. 2017, 3, 29.

[10] Y. Y. Kim , T.‐Y. Yang , R. Suhonen , A. Kemppainen , K. Hwang , N. J. Jeon , J. Seo , Nat. Commun. 2020, 11, 5146. 33051454 10.1038/s41467-020-18940-5 PMC7555830

[11] Y. Y. Kim , T.‐Y. Yang , R. Suhonen , M. Välimäki , T. Maaninen , A. Kemppainen , N. J. Jeon , J. Seo , Adv. Sci. 2019, 6, 1802094. 10.1002/advs.201802094 PMC6446604 30989030

[12] A. J. L. Garcia , G. Sico , M. Montanino , V. Defoor , M. Pusty , X. Mescot , F. Loffredo , F. Villani , G. Nenna , G. Ardila , Nanomaterials 2021, 11, 1430. 34071555 10.3390/nano11061430 PMC8226623

[13] C. Chen , J. Chen , H. Han , L. Chao , J. Hu , T. Niu , H. Dong , S. Yang , Y. Xia , Y. Chen , W. Huang , Nature 2022, 612, 266. 36352221 10.1038/s41586-022-05346-0

[14] P. He , J. Cao , H. Ding , C. Liu , J. Neilson , Z. Li , I. A. Kinloch , B. Derby , ACS Appl. Mater. Interfaces 2019, 11, 32225. 31390171 10.1021/acsami.9b04589

[15] I. Brunetti , L. Pimpolari , S. Conti , R. Worsley , S. Majee , D. K. Polyushkin , M. Paur , E. Dimaggio , G. Pennelli , G. Iannaccone , M. Macucci , F. Pieri , T. Mueller , C. Casiraghi , G. Fiori , npj 2D Mater. Appl. 2021, 5, 85.

[16] Y. Wang , C. Yan , S.‐Y. Cheng , Z.‐Q. Xu , X. Sun , Y.‐H. Xu , J.‐J. Chen , Z. Jiang , K. Liang , Z.‐S. Feng , Adv. Funct. Mater. 2019, 29, 1902579.

[17] P. Andersson , D. Nilsson , P.‐O. Svensson , M. Chen , A. Malmström , T. Remonen , T. Kugler , M. Berggren , Adv. Mater. 2002, 14, 1460.

[18] R. Mannerbro , M. Ranlöf , N. Robinson , R. Forchheimer , Synth. Met. 2008, 158, 556.

[19] D. McManus , S. Vranic , F. Withers , V. Sanchez‐Romaguera , M. Macucci , H. Yang , R. Sorrentino , K. Parvez , S.‐K. Son , G. Iannaccone , K. Kostarelos , G. Fiori , C. Casiraghi , Nat. Nanotechnol. 2017, 12, 343. 28135260 10.1038/nnano.2016.281

[20] C. Trudeau , P. Beaupré , M. Bolduc , S. G. Cloutier , npj Flexible Electron. 2020, 4, 34.

[21] S. Chung , S. O. Kim , S.‐K. Kwon , C. Lee , Y. Hong , IEEE Electron Device Lett. 2011, 32, 1134.

[22] T. Carey , S. Cacovich , G. Divitini , J. Ren , A. Mansouri , J. M. Kim , C. Wang , C. Ducati , R. Sordan , F. Torrisi , Nat. Commun. 2017, 8, 1202. 29089495 10.1038/s41467-017-01210-2 PMC5663939

[23] W. G. Whittow , A. Chauraya , J. C. Vardaxoglou , Y. Li , R. Torah , K. Yang , S. Beeby , J. Tudor , IEEE Antennas Wireless Propag. Lett. 2014, 13, 71.

[24] F. Molina‐Lopez , T. Z. Gao , U. Kraft , C. Zhu , T. Öhlund , R. Pfattner , V. R. Feig , Y. Kim , S. Wang , Y. Yun , Z. Bao , Nat. Commun. 2019, 10, 2676. 31213599 10.1038/s41467-019-10569-3 PMC6582140

[25] C. Ye , M. Wang , J. Min , R. Y. Tay , H. Lukas , J. R. Sempionatto , J. Li , C. Xu , W. Gao , Nat. Nanotechnol. 2023, 19, 330. 37770648 10.1038/s41565-023-01513-0 PMC10954395

[26] H. J. Park , J. Jeong , S. G. Son , S. J. Kim , M. Lee , H. J. Kim , J. Jeong , S. Y. Hwang , J. Park , Y. Eom , B. G. Choi , Adv. Funct. Mater. 2021, 31, 2011059.

[27] V. Subramanian , J. Cen , A. de la Fuente Vornbrock , G. Grau , H. Kang , R. Kitsomboonloha , D. Soltman , H.‐Y. Tseng , Proc. IEEE 2015, 103, 567.

[28] Z. Lin , Y. Liu , U. Halim , M. Ding , Y. Liu , Y. Wang , C. Jia , P. Chen , X. Duan , C. Wang , F. Song , M. Li , C. Wan , Y. Huang , X. Duan , Nature 2018, 562, 254. 30283139 10.1038/s41586-018-0574-4

[29] E. Ramon , E. Sowade , C. Martinez‐Domingo , K. Y. Mitra , A. Alcalde , R. R. Baumann , J. Carrabina , Flexible Printed Electron. 2021, 6, 15003.

[30] D. Tobjörk , R. Österbacka , Adv. Mater. 2011, 23, 1935. 21433116 10.1002/adma.201004692

[31] Y. Zhang , L. Zhang , K. Cui , S. Ge , X. Cheng , M. Yan , J. Yu , H. Liu , Adv. Mater. 2018, 30, 1801588. 10.1002/adma.201801588 30066444

[32] T. Pinheiro , R. Correia , M. Morais , J. Coelho , E. Fortunato , M. G. F. Sales , A. C. Marques , R. Martins , ACS Nano 2022, 16, 20633. 36383513 10.1021/acsnano.2c07596 PMC9798867

[33] E. Mahmoodi , M. H. Amiri , A. Salimi , R. Frisenda , E. Flores , J. R. Ares , I. J. Ferrer , A. Castellanos‐Gomez , F. Ghasemi , Sci. Rep. 2022, 12, 12585. 35869156 10.1038/s41598-022-16834-8 PMC9307754

[34] B. Nouri , A. Castellanos‐Gomez , F. Ghasemi , J. Alloys Compd. 2023, 959, 170554.

[35] W. Zhang , O. Çakıroğlu , A. Al‐Enizi , A. Nafady , X. Gan , X. Ma , S. Kuriakose , Y. Xie , A. Castellanos‐Gomez , Opto‐electron. Adv. 2023, 6, 220101.

[36] A. Mazaheri , M. Lee , H. S. J. van der Zant , R. Frisenda , A. Castellanos‐Gomez , Nanoscale 2020, 12, 19068. 32568333 10.1039/d0nr02268c

[37] J. Zikulnig , C. Hirschl , L. Rauter , M. Krivec , H. Lammer , F. Riemelmoser , A. Roshanghias , Flexible Printed Electron. 2019, 4, 15008.

[38] S. H. Ferreira , I. Cunha , J. V. Pinto , J. P. Neto , L. Pereira , E. Fortunato , R. Martins , Chemosensors 2021, 9, 192.

[39] I. Cunha , J. Martins , P. G. Bahubalindruni , J. T. Carvalho , J. Rodrigues , S. Rubin , E. Fortunato , R. Martins , L. Pereira , Adv. Mater. Technol. 2021, 6, 2100633.

[40] N. Maldonado , V. G. Vegas , O. Halevi , J. I. Martínez , P. S. Lee , S. Magdassi , M. T. Wharmby , A. E. Platero‐Prats , C. Moreno , F. Zamora , P. Amo‐Ochoa , Adv. Funct. Mater. 2019, 29, 1808424.

[41] Z. Gozutok , O. Kinj , I. Torun , A. T. Ozdemir , M. S. Onses , Cellulose 2019, 26, 3503.

[42] L. Hu , H. Wu , F. La Mantia , Y. Yang , Y. Cui , ACS Nano 2010, 4, 5843. 20836501 10.1021/nn1018158

[43] G. Ersu , Y. Sozen , E. Sánchez‐Viso , S. Kuriakose , B. H. Juárez , F. J. Mompean , M. Garcia‐Hernandez , L. Visscher , A. J. Magdaleno , F. Prins , A. M. Al‐Enizi , A. Nafady , C. Munuera , J. O. Island , A. Castellanos‐Gomez , Adv. Eng. Mater. 2023, 25, 2300226.

[44] M. A. Pimenta , G. Dresselhaus , M. S. Dresselhaus , L. G. Cançado , A. Jorio , R. Saito , Phys. Chem. Chem. Phys. 2007, 9, 1276. 17347700 10.1039/b613962k

[45] R. Escribano , J. J. Sloan , N. Siddique , N. Sze , T. Dudev , Vib. Spectrosc. 2001, 26, 179.

[46] Z. Lin , P. Karthik , M. Hada , T. Nishikawa , Y. Hayashi , Nanomaterials 2017, 7, 125. 28555015 10.3390/nano7060125 PMC5485772

[47] V. Forsberg , J. Mašlík , M. Norgren , RSC Adv. 2019, 9, 23925. 35530632 10.1039/c9ra03801a PMC9069492

[48] H. Lee , Y. Kim , H. Cho , J. Lee , J. H. Kim , RSC Adv. 2019, 9, 17318. 35519849 10.1039/c9ra03040a PMC9064581

[49] J. Quereda , Q. Zhao , E. Diez , R. Frisenda , A. Castellanos‐Gomez , Open Res. Eur. 2021, 1, 98. 37645138 10.12688/openreseurope.14018.2 PMC10446081

[50] K. Lobo , R. Thakur , S. K. Prasad , H. S. S. R. Matte , J. Mater. Chem. C 2022, 10, 18326.

[51] T. Leng , K. Parvez , K. Pan , J. Ali , D. McManus , K. S. Novoselov , C. Casiraghi , Z. Hu , 2D Mater. 2020, 7, 024004.

[52] C.‐H. Lin , D.‐S. Tsai , T.‐C. Wei , D.‐H. Lien , J.‐J. Ke , C.‐H. Su , J.‐Y. Sun , Y.‐C. Liao , J.‐H. He , ACS Nano 2017, 11, 10230. 28945959 10.1021/acsnano.7b04804

[53] D. McManus , A. Dal Santo , P. B. Selvasundaram , R. Krupke , A. LiBassi , C. Casiraghi , Flexible Printed Electron. 2018, 3, 034005.

[54] D. Kufer , G. Konstantatos , Nano Lett. 2015, 15, 7307. 26501356 10.1021/acs.nanolett.5b02559

[55] J. O. Island , S. I. Blanter , M. Buscema , H. S. J. van der Zant , A. Castellanos‐Gomez , Nano Lett. 2015, 15, 7853. 26540135 10.1021/acs.nanolett.5b02523

[56] M. M. Furchi , D. K. Polyushkin , A. Pospischil , T. Mueller , Nano Lett. 2014, 14, 6165. 25299515 10.1021/nl502339q

[57] A. C. Ugural , in Mechanics of Materials, Wiley, New York 2008.

[58] J. Jiang , J. Sun , W. Dou , B. Zhou , Q. Wan , Appl. Phys. Lett. 2011, 98, 113507.

[59] R. Martins , D. Gaspar , M. J. Mendes , L. Pereira , J. Martins , P. Bahubalindruni , P. Barquinha , E. Fortunato , Appl. Phys. Lett. 2018, 12, 402.

[60] D. Gaspar , J. Martins , P. Bahubalindruni , L. Pereira , E. Fortunato , R. Martins , Adv. Electron. Mater. 2018, 4, 1800423.

[61] W. Zhang , Q. Zhao , C. Munuera , M. Lee , E. Flores , J. E. F. Rodrigues , J. R. Ares , C. Sanchez , J. Gainza , H. S. J. van der Zant , J. A. Alonso , I. J. Ferrer , T. Wang , R. Frisenda , A. Castellanos‐Gomez , Appl. Mater. Today 2021, 23, 101012.

[62] Z. Liu , S. Nie , J. Luo , Y. Gao , X. Wang , Q. Wan , Adv. Electron. Mater. 2019, 5, 1900235.

[63] F. Bonafè , F. Decataldo , B. Fraboni , T. Cramer , Adv. Electron. Mater. 2021, 7, 2100086.

[64] K. S. Reddy , S. Veeralingam , P. H. Borse , S. Badhulika , Org. Electron. 2022, 108, 106586.

[65] J. Hao , L. Wang , Y. Ma , P. Zeng , L. Guo , W. Wang , G. Liu , Semicond. Sci. Technol. 2020, 35, 085016.

[66] M.‐L. Tsai , D.‐S. Tsai , L. Tang , L.‐J. Chen , S. P. Lau , J.‐H. He , ACS Nano 2017, 11, 4564. 28430415 10.1021/acsnano.6b08567

[67] D. Zhang , W. Zheng , R. Lin , Y. Li , F. Huang , Adv. Funct. Mater. 2019, 29, 1900935.

