# Supplementary material for: Flexible Phototransistors on Paper: Scalable Fabrication of PEDOT:PSS Devices Using a Pen Plotter
Source: Small Sci. 2024 Sep 16;4(11):2400063. doi: 10.1002/smsc.202400063 (PMC11935257; doi:10.1002/smsc.202400063)
Supplement: Supplementary file 1 — Supplementary Material [file SMSC-4-2400063-s001.pdf]

Supporting Information:

Flexible phototransistors on paper: scalable fabrication of PEDOT:PSS devices using a pen plotter

Y. Sozen[1,], G. Ersu[1], T. Pucher[1], J. Quereda[1] and A. Castellanos-Gomez[1,*]*

[1]Materials Science Factory. Instituto de Ciencia de Materiales de Madrid (ICMM-CSIC), Madrid, E-28049, Spain.

yigit.sozen@csic.es, andres.castellanos@csic.es

Possible failure mechanisms and durability assessment

Understanding the possible failure mechanisms that may be encountered during the large-batch fabrication process is important to enhance the reliability and the efficiency of our method. Based on our observations, one of the most important factors that can cause device malfunction or a decrease or variation in device performance is the flow rate of the ink through the marker pen during the plotting. The flow characteristics of an ink change depending on its composition. Higher viscosity inks will tend to flow more slowly, while lower viscosity inks will tend to flow more quickly from the tip of the pen. At this point, one of the most important parameters to choose carefully is the print speed. This parameter allows us to adjust the contact time of the pen with the paper surface, thus controlling the amount of ink deposited per time. Accordingly, it is important to work with low (high) printing speeds for the inks with high (low) viscosity. Determining an optimal printing speed depending on the ink viscosity will help to achieve more uniform patterns, precise printing, and avoid damage to the paper surface. Although viscosity is clearly a factor one can play with, we decided to use both graphite and PEDOT:PSS commercial inks thus fixing this parameter in our experiments. In our previous study, we reported that printing speeds between 300 and 1200 mm min^{-1} are suitable depending on the viscosity of these ink.[43] Ink flow is also affected by the size and shape of the pen tip. Differences in tip shape can result in differences in flow rate

and line width. This determines how much ink is dispensed onto the paper surface during the plotting. Another factor that affects the flow rate is the pressure applied to the pen against the paper surface. Too much pressure could cause an excessive flow of the ink and also could abrade the paper surface. In our previous study, we reported that applying a force of 1.2 N is appropriate during the plotting process.[43] Finally, clogging of the marker tip can also change the flow rate, resulting in reduced or irregular ink flow through the tip. The most common reason is that the ink can dry out when marker pen is not used over a long time. A method to handle this problem is removing the tip and rubbing it with an isopropanol or ethanol, which help to remove the dried ink making the marker usable again.

We faced several failure scenarios during the fabrication depending on the high and low rate flow of the ink. For example, graphite ink with a high flow rate spreads more onto the paper surface and results in short between the electrodes. In addition, this diffusion can cause large variations in channel length, resulting in non-uniform device characteristics. To avoid from this scenario, one should determine the optimal printing speed and pressure for the ink will be used. We also obtain that during the long plotting process, the flow rate can drop owing to the least ink flow to the pen tip over time. This usually occurs using the deformed or clogged pen tip, resulting in incomplete or less coverage, leading to poor device performance and inconsistent characteristics between devices. Therefore, for the long-duration printing, it is important to use a well-filled marker with a new tip.

Channel-length dependent electronic properties

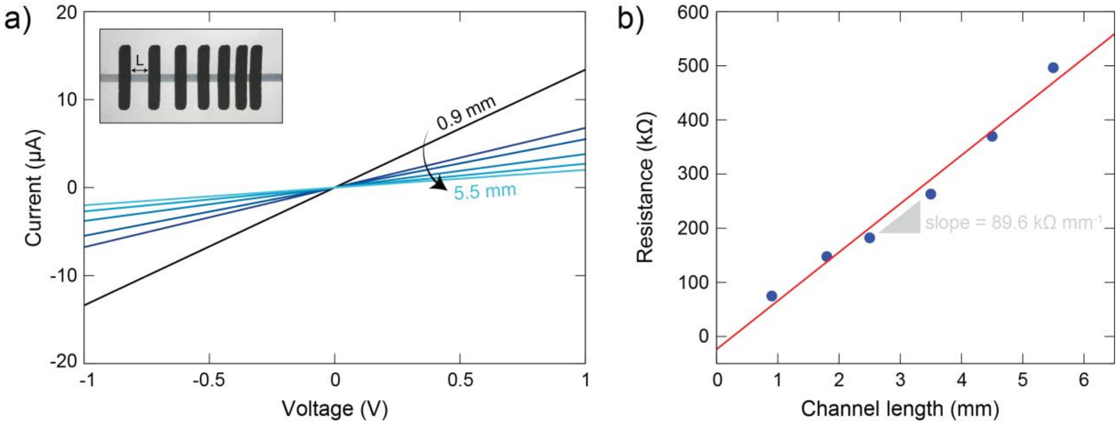

Figure S1. (a) Current versus voltage curves for PEDOT:PSS channel with varying channel lengths. The inset image shows the deposited graphite electrodes on PEDOT:PSS with varied channel lengths. (b) Extracted resistance values from the *IV* curves represented in (a).

Strain- dependent photocurrent characteristics

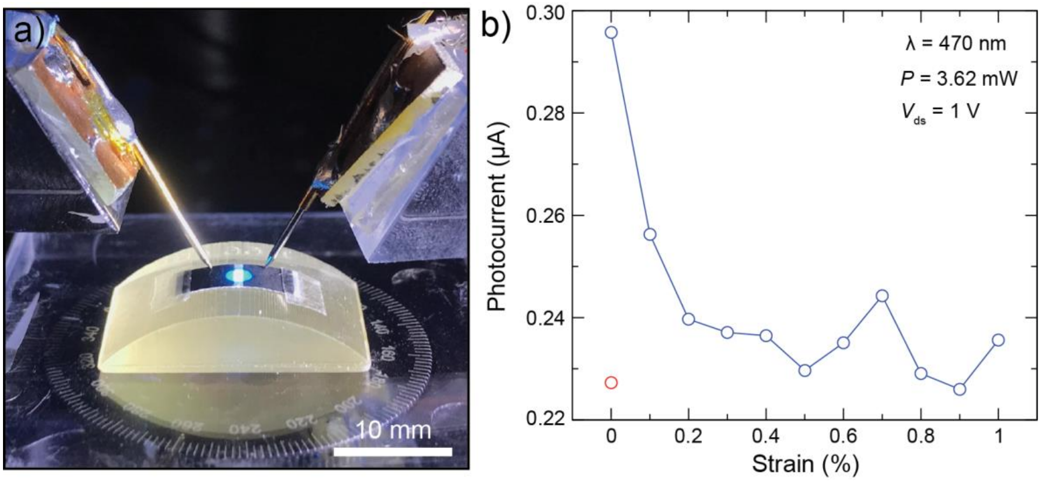

Figure S2. (a) The uniaxial strained image of the device under illumination. (b) Measured photocurrent values with varying strain levels from 0 to 1 %. The red dot corresponds to the photocurrent data collected after strain measurement.

Stability of the devices under atmospheric conditions

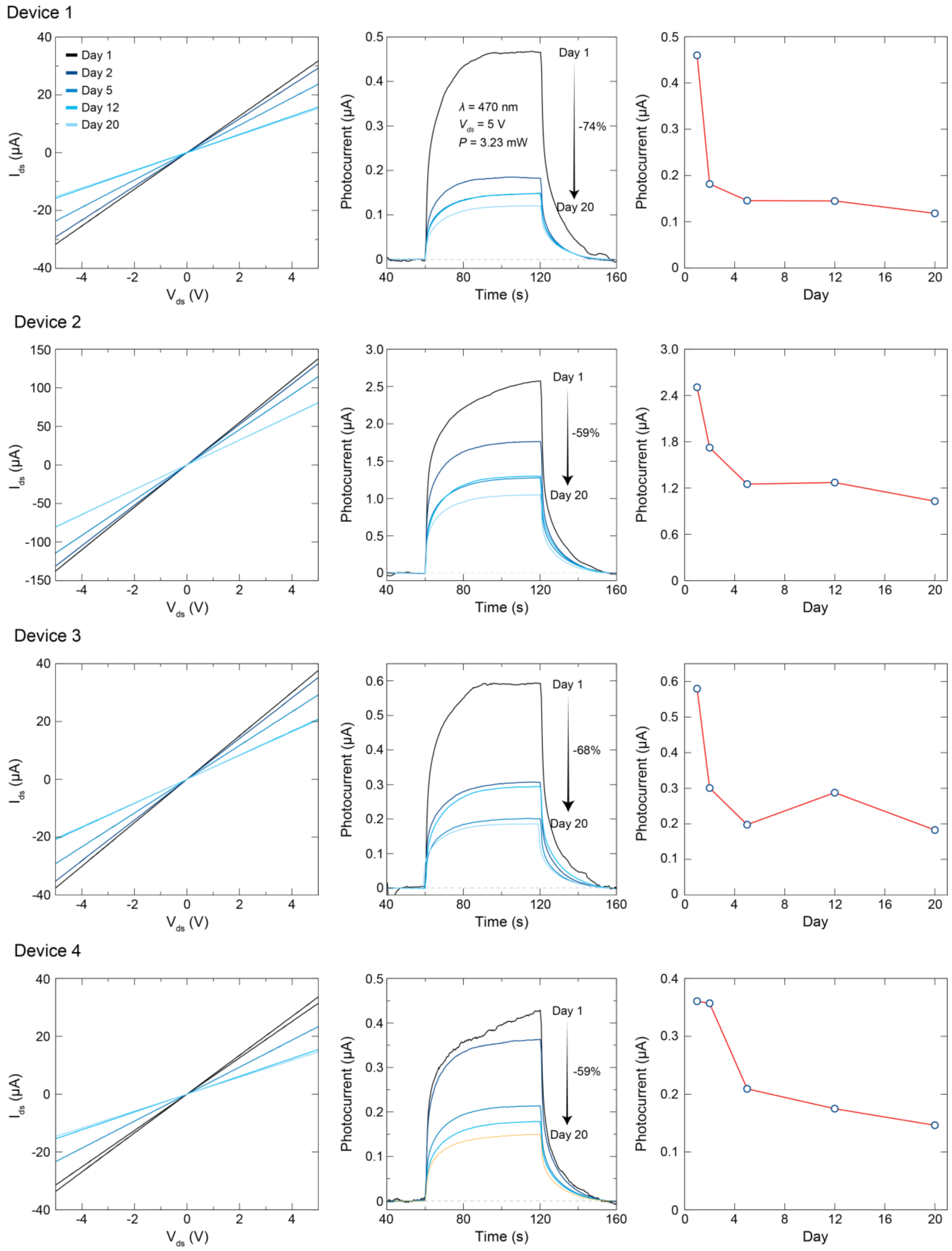

Figure S3. Left, mid and, right columns represents the evolution of *IV* curves, time-resolved photocurrent measurements, and photocurrent values, respectively, for 4 different PEDOT:PSS/graphite devices under atmospheric conditions over 20 days.

Stability under continuous photoswitching

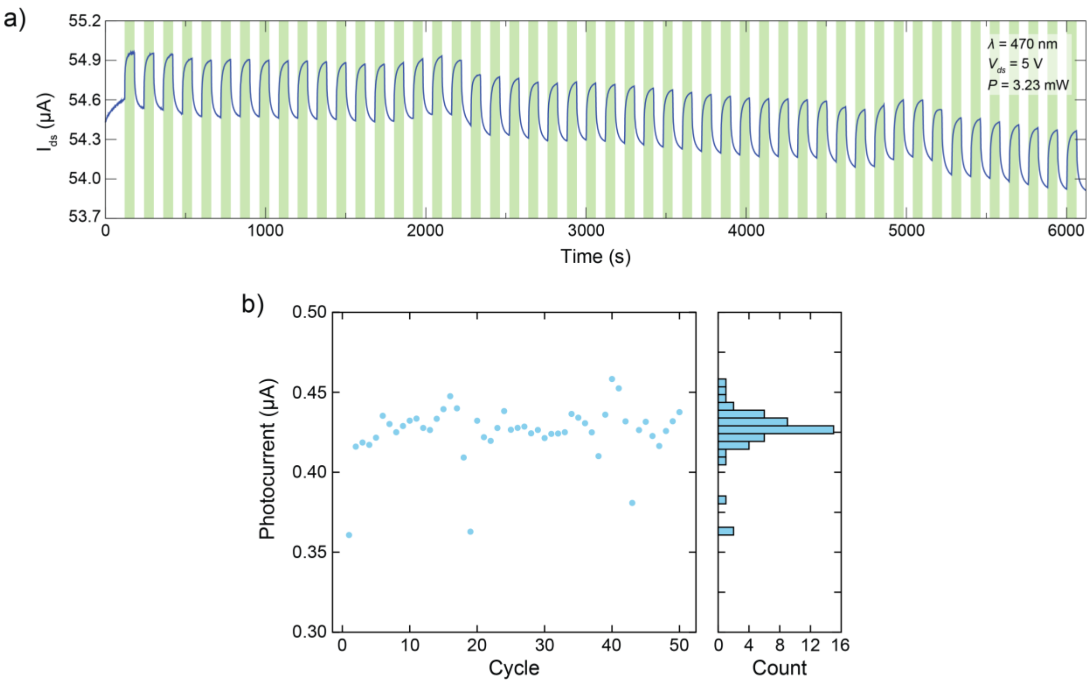

Figure S4. (a) Current vs time measurement while PEDOT:PSS/graphite photodetector is irradiated with 50 cycles photoswitching. (b) Photocurrent values obtained from each ON/OFF cycle. 1D histogram gives the photocurrent distribution.

Temperature dependent photocurrent measurements

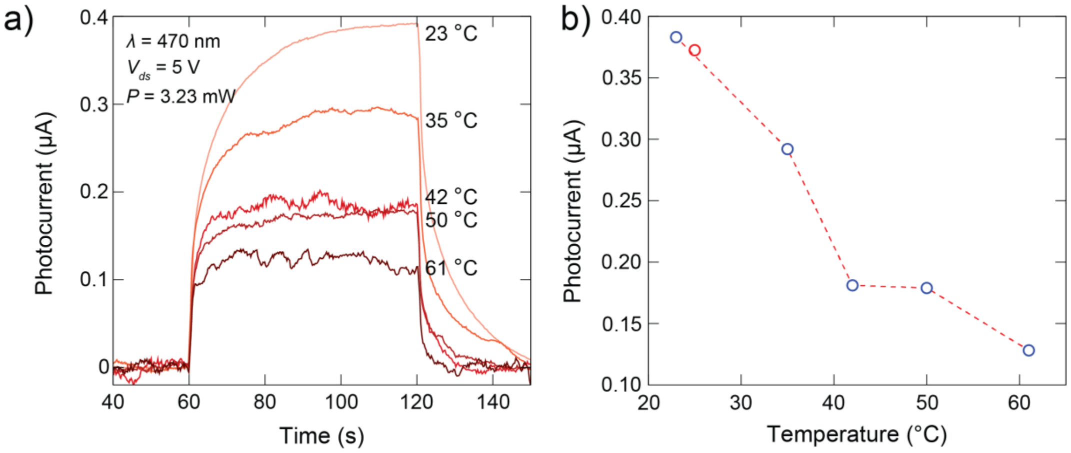

Figure S5. (a) The evolution of photocurrent time traces with increasing temperature. (b) Photocurrent values as a function of the temperature. Red dotted data represents the photocurrent value obtained at room temperature after temperature dependent measurements.

Images from PEDOT:PSS/graphite transistor

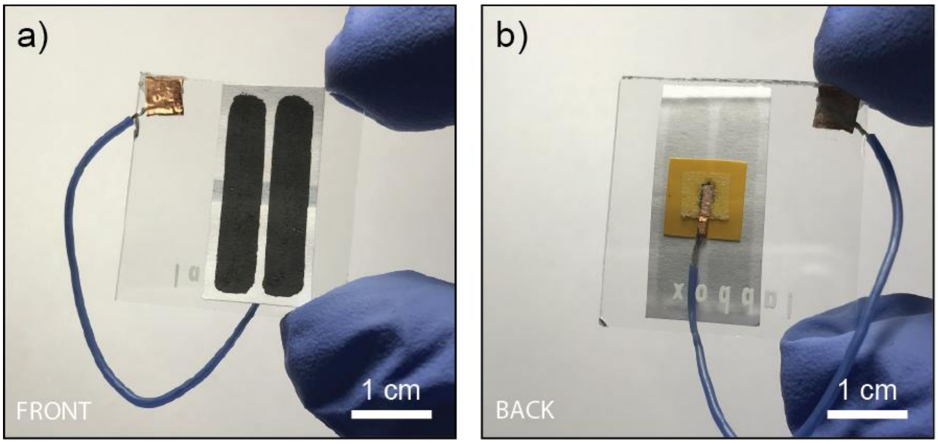

Figure S6. (a) Front and (b) back views of the PEDOT:PSS/graphite transistor replaced on a glass slide to perform gate-dependent electronic measurements.

Electronic properties of back-gated PEDOT:PSS/graphite for two different samples

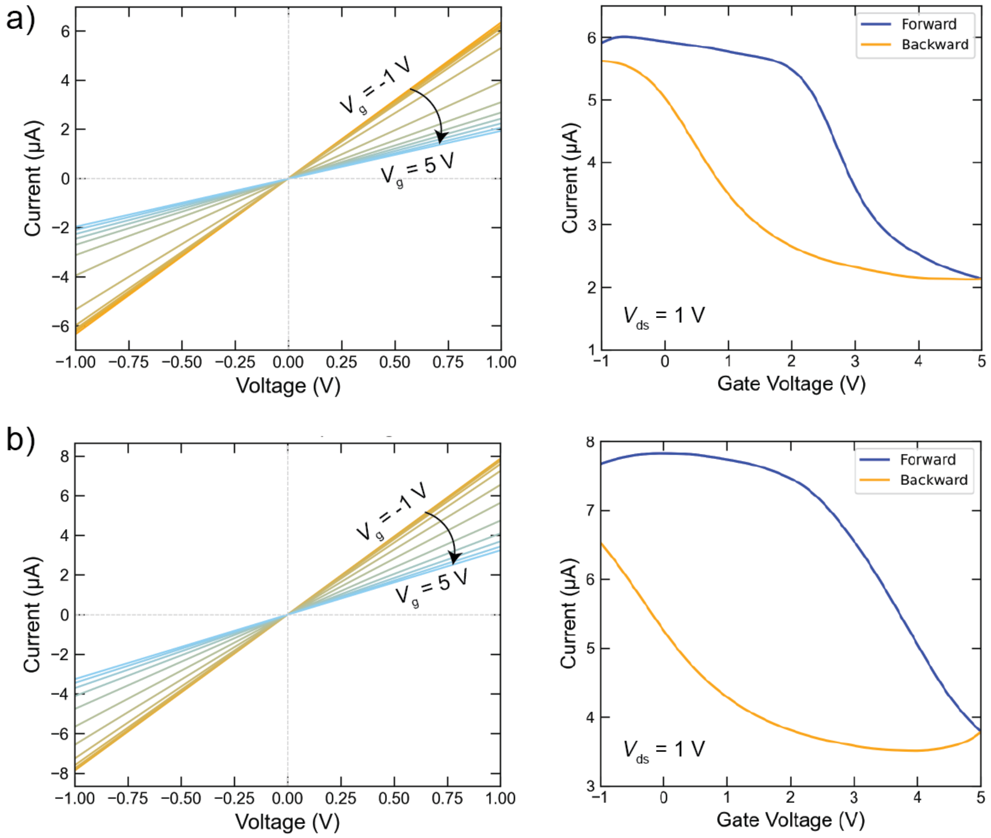

Figure S7. The left and right panels in (a) and (b) represent gate-dependent *IV* characteristics, and transfer curves, respectively, obtained from two different PEDOT:PSS/graphite devices.

The evaluation of photocurrent as a function of the gate voltage

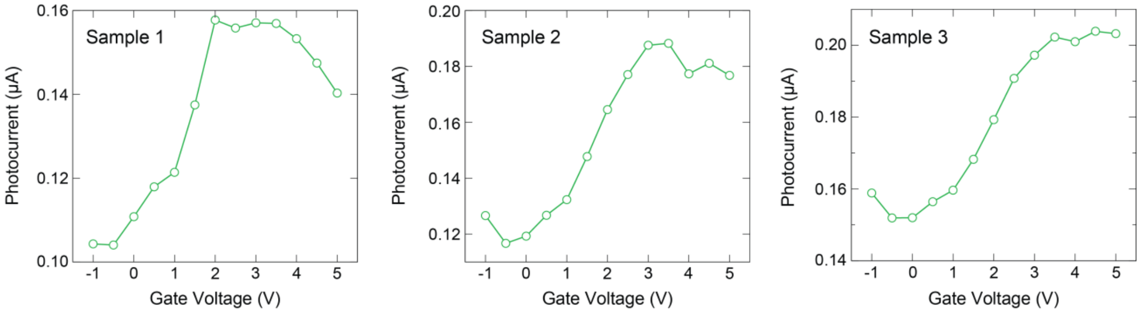

Figure S8. Photocurrent values measured under different gate voltages for 3 different samples.

Gate-dependent photocurrent features of sample 2

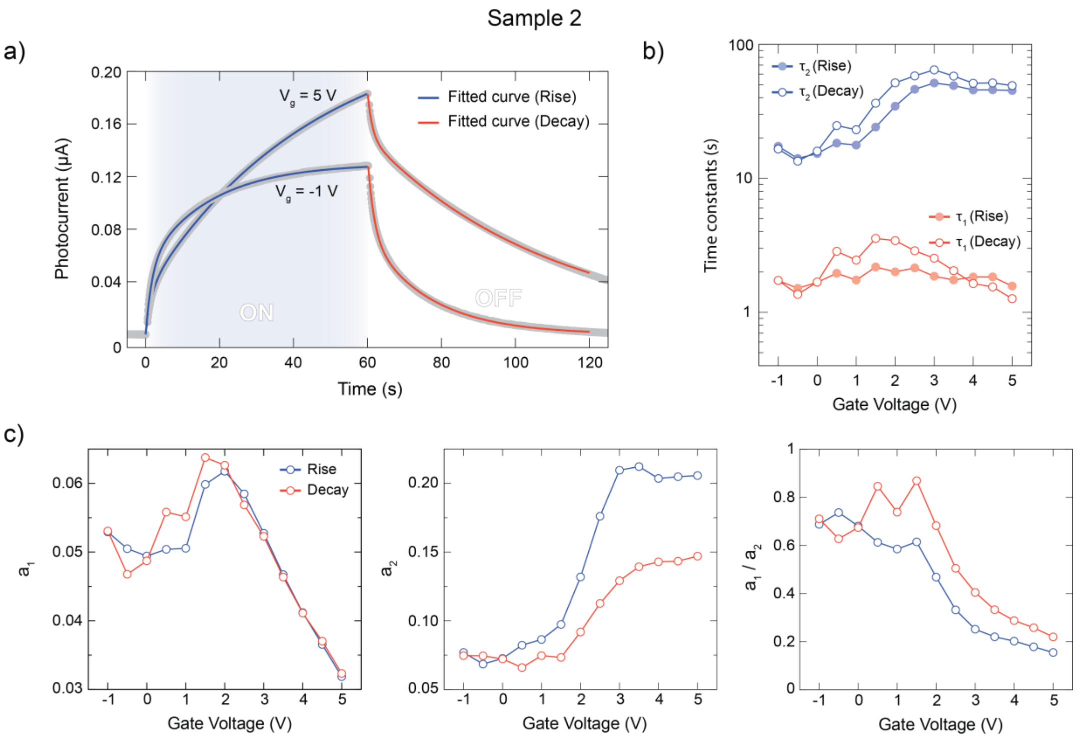

Figure S9. (a) Photocurrent time traces obtained at two different gate voltages. Blue and red curves are two-term exponential fit to the rising and decaying part of the corresponding data. Gate-dependent change in (b) time constants and (c) amplitudes derived from two-term exponential fit performed for rising and decaying signals. (c) The ratio of the amplitudes. Measurement is performed by using 470 nm light with 3.62 mW power, under a bias voltage of 1 V.

Gate-dependent photocurrent features of sample 3

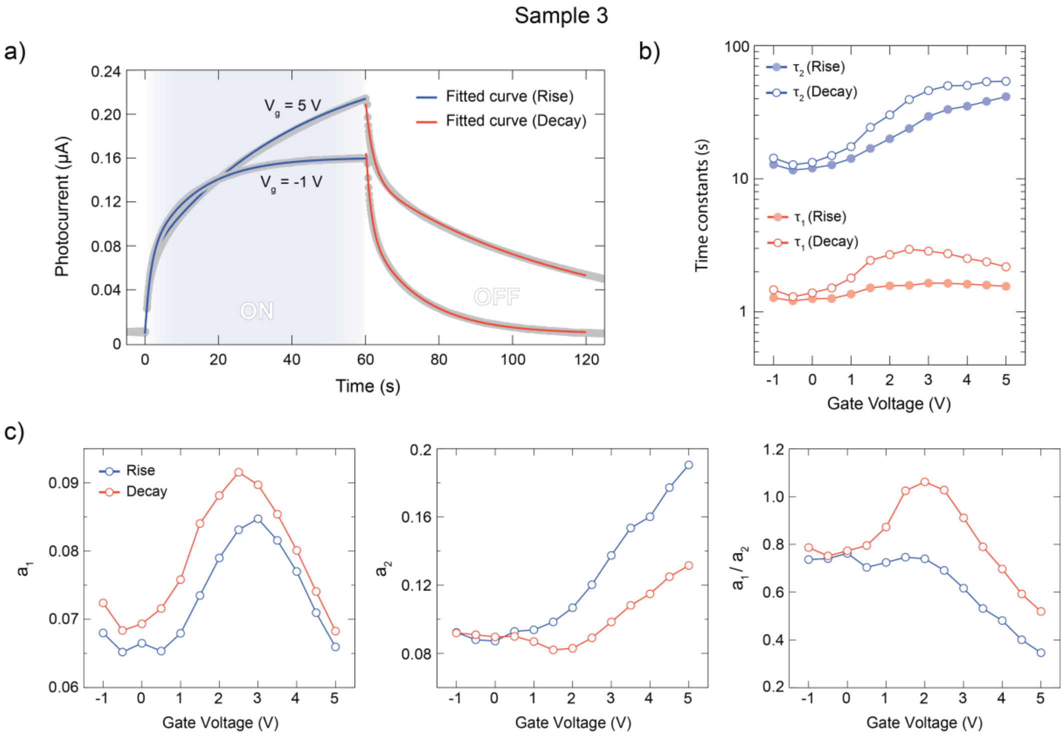

Figure S10. (a) Photocurrent time traces obtained at two different gate voltages. Blue and red curves are two-term exponential fit to the rising and decaying part of the corresponding data. Gate-dependent change in (b) time constants and (c) amplitudes derived from two-term exponential fit performed for rising and decaying signals. (c) The ratio of the amplitudes. Measurement is performed by using 470 nm light with 3.62 mW power, under a bias voltage of 1 V.
